# The Natural History and Clinical Presentation of Cervical Spondylotic Myelopathy

**DOI:** 10.1155/2012/480643

**Published:** 2011-12-22

**Authors:** Chester K. Yarbrough, Rory K. J. Murphy, Wilson Z. Ray, Todd J. Stewart

**Affiliations:** Department of Neurosurgery, Washington University School of Medicine, St. Louis, MO 63110, USA

## Abstract

Cervical spondylotic myelopathy (CSM) refers to impaired function of the spinal cord caused by degenerative changes of the cervical spine resulting in spinal cord compression. It is the most common disorder in the United States causing dysfunction of the spinal cord. A literature review of the natural history of mild cervical myelopathy is undertaken. Clinical presentation and current concepts of pathophysiology are also discussed. While many patients with mild signs of CSM will stabilize or improve over time with conservative treatment, the clinical course of a specific individual patient cannot be predicted. Asymptomatic patients with cervical stenosis and abnormalities on electrophysiologic studies may be at higher risk for developing myelopathy.

## 1. Natural History of Mild Cervical Spondylotic Myelopathy

Cervical spondylosis refers to osteoarthritic degeneration of the cervical spine. Brain et al. suggested symptomatology, whether radiculopathy or myelopathy, resulted from disc protrusion and associated soft tissue abnormalities [1, 2]. Although degeneration can occur secondary to various causes, years of motion and activity, commonly referred to as “wear and tear,” is the most common etiology. Several studies have shown in animal models and in humans that excessive motion and repetitive micro-trauma accelerates degenerative changes [3–9]. The accumulation of degenerative changes affects both canal diameter and sagittal mobility of the cervical spine [10]. Additionally, a congenitally narrow spinal canal may predispose one to formation of CSM [11–14]. In current understanding, cervical spondylosis encompasses degenerative changes affecting the uncovertebral joints, facet joints, intervertebral discs, and the other soft tissue and bony components of the cervical spine. While it may affect only a single level, spondylosis has been shown to commonly begin at lower levels with subsequent progressive involvement of multiple spinal levels [15].

While the recommendation for surgical treatment of patients with severe, progressive myelopathy seems straightforward, it is less clear how to properly manage patients with cervical spondylosis and very subtle signs of myelopathy. Several authors have described the clinical course of patients with symptomatic cervical spondylosis. Initial authors supported clinical stability in this patient population. Clarke and Robinson retrospectively described 120 patients with CSM, 26 of whom were treated conservatively [16]. Nearly 80% of these patients presented with weakness or sensory loss in one or more limbs, while 18% presented with pain. Clarke and Robinson showed that approximately 75% of patients showed episodic progression of symptoms with intervening stability, though approximately two-thirds of patients showed subtle clinical decline during periods of stability. In 20% of patients, slow and steady deterioration occurred. In 5%, onset of symptoms and signs was followed by a long period of stability without any additional deterioration. Overall, approximately half of the conservatively managed patients improved at some point in the clinical course [16]. Lees and Turner described 44 patients with CSM and 51 patients with spondylosis without myelopathy [17]. Of the 44 patients with CSM, 28 patients were managed conservatively with a cervical collar, with 17 showing improvement over time [17]. In contrast to the authors listed above, several other groups have suggested that CSM has a largely progressive course over time [18, 19]. Matsumoto et al. described a case series in which one-third of patients with mild CSM had progression of symptoms while undergoing conservative management [20]. Sadasivan et al. reported 22 patients with CSM of several years duration, all of whom suffered clinical progression of disease over time [21]. It is important to note that the studies described above tended towards patients with mild and moderate disease processes, although some severely affected individuals were also included.

With the creation and subsequent modification of the Japanese Orthopedic Association score for myelopathy [22–24], a statistically valid and reproducible method of assessing CSM allowed further characterization of this patient population. Kada ňka et al. suggested that 80% of patients with mild myelopathy will improve with or without surgery [25, 26]. Shimomura et al. produced similar results, with 80% of patients showing clinically stable myelopathy over a 3-year period [27]. Other authors have found similar results with conservative management [28, 29]. However, subjective self-assessment and general health may decline over time, affecting the recommendation of conservative versus surgical intervention [30].

Because of the varied nature of progression in mild myelopathy, several authors have investigated other methods for identifying patients with either cervical spondylosis or mild cervical spondylotic myelopathy with higher risk of progression to moderate or severe myelopathy. Asymptomatic spondylotic patients with abnormal somatosensory evoked potentials and radiculopathy have shown increased propensity to progress towards clinical myelopathy [31–34]. Interestingly, in their study regarding electrophysiologic findings affecting progression from asymptomatic stenosis to CSM, the degree of compression as measured by the anterior-posterior diameter divided by transverse diameter did not affect development of CSM [32].

Review of the literature shows that the clinical course of cervical myelopathy is variable and that conservative management may result in stability or improvement of symptoms in the majority of patients with mild symptoms [25–29]. Predicting the clinical course of a single patient remains difficult, though some evidence suggests that younger patients and those with mild symptoms are more likely to improve [35].

## 2. Pathology of CSM

Several authors have described pathologic findings associated with CSM in cadaveric studies of patients with CSM [36–38]. Pathologic findings include atrophy, neuronal loss in gray matter, and demyelination in the surrounding white matter. Interestingly, these findings are similar to those found in patients with transient hypoperfusion. The magnitude of pathologic findings correlates with the length of myelopathy and directly relates to the degree of canal stenosis [36–38]. Several authors have found that imaging findings including diffusion tensor imaging and apparent diffusion coefficient maps also show white-matter tract changes at the corresponding levels of compression [39–41].

## 3. Clinical Presentations

CSM may present with divergent clinical findings depending on the levels affected, involvement of the neural foramina, and long tract involvement. A variety of neurological signs and symptoms may be present, including sensory changes, reflex abnormalities, decreased dexterity, weakness, gait instability, bowel and bladder dysfunction, spasticity, presence of Hoffman's and/or Babinski's sign, axial neck pain, radiculopathy, and even acute spinal cord injury [42–44]. The variation in symptoms caused by involvement of the various cervical levels results in a large possibility of clinical presentations affecting almost any muscle of the body.

Some authors have attempted to distinguish the various presentations into a categorization schema. Crandall and Batzdorf suggested clinical grounds for classifying patients into transverse lesion syndrome, motor system syndrome, central cord syndrome, Brown-Sequard syndrome, and brachialgia and cord syndrome [45]. Other authors have separated the varying presentations: anatomic involvement, with a lateral or radicular syndrome, medial or myelopathy syndrome, a combined medial and lateral syndrome, a vascular syndrome, and an anterior syndrome [46, 47]. Most frequently, clinicians rely on clinical signs and symptoms of myelopathy rather than the syndrome names above to describe a patient's condition. Severity of symptoms, functional impairment, and progression of symptoms rather than clinical syndrome classification drive decision-making for therapeutic interventions.

As radiographic studies have improved and expanded in use, more patients will likely come for evaluation with radiographic diagnosis of cervical stenosis. Secondary to the explosion of imaging technology and utilization, the patient population seen in spine clinics today may represent a slightly different population than in the past. Given the variability of symptom progression, clinical experience and care should guide management of these patients towards conservative management.

## 4. Conclusion

Cervical spondylotic myelopathy occurs in age-dependent fashion as degenerative changes occur in the cervical spinal cord. Presenting signs and symptoms are highly variable and may stabilize or improve over time with conservative management. Abnormal electrophysiology and presence of radiculopathy may portend an increased chance of progression from asymptomatic cervical spondylosis to myelopathy.

## Abbreviations

CSM: Cervical Spondylotic Myelopathy.
